# Split feeding for laying hens: a step beyond precision nutrition

**DOI:** 10.1016/j.psj.2025.105158

**Published:** 2025-04-13

**Authors:** Carlos Henrique do Nascimento, Adiel Vieira de Lima, Paloma Eduarda Lopes de Souza, Djalma Fernandes de Souza Filho, Raiane dos Santos Silva, Aline Beatriz Rodrigues, Raul da Cunha Lima Neto, Matheus Ramalho de Lima, Ricardo Romão Guerra, Apolônio Gomes Ribeiro, Lucas Rannier Ribeiro Antonino Carvalho, Fernando Guilherme Perazzo Costa

**Affiliations:** a Universidade Federal da Paraíba, Animal Science Department, Areia-PB, Brazil; b Universidade Federal do Rio Grande do Norte, Animal Science Departament, Unidade Acadêmica Especializada em Ciências Agrarias, Macaíba - RN, Brazil; c Universidade Federal do Oeste do Pará – Instituto de Biodiversidade e Floresta, Santarém - PA, Brazil; d Universidade Federal Rural do Semi-Árido, Animal Science Department, Mossoró - RN, Brazil; e Department of Physiology and Pharmacology Karolinska Institutet - Stockholm Sweden Biomedicum, 5B, Solnavägen 9 S-171 77, Stockholm, Sweden

**Keywords:** Calcium carbonate, Egg quality, Feeding Behavior, Feeding management, Physiological Needs

## Abstract

The primary objective of modern laying hen production is to extend the production cycle to achieve an average of 500 eggs in 100 weeks, which requires research to improve eggshell quality. The eggshell plays a crucial role in mechanical protection and provides a suitable environment for embryonic development. Composed mainly of calcium carbonate and other minerals, calcium (Ca) is essential in the diet for eggshell formation and is incorporated into the diet of laying hens. However, hens have a cyclic reproductive physiology, which leads to varying nutrient requirements throughout the day. In the morning, higher levels of dietary protein and energy are needed to support yolk and albumen formation, while in the afternoon and evening, increased calcium intake is crucial for eggshell and membrane development. Traditional feeding systems, which provide a single diet throughout the day, may result in nutrient imbalances, leading to excess calcium intake in the morning and an oversupply of protein and energy in the afternoon. To address this issue, the split feeding strategy has been proposed. This approach involves offering a high-protein, high-energy, low-calcium diet in the morning, followed by a low-protein, low-energy, high-calcium diet in the afternoon or evening. By aligning nutrient supply with the hen's metabolic needs, split feeding optimizes nutrient utilization, improves feed efficiency, and enhances eggshell quality while reducing unnecessary nutrient excretion. Given the growing interest in optimizing layer nutrition and improving production efficiency, this study presents a comprehensive review of existing data on split feeding strategies, highlighting their impact on nutrient utilization, eggshell quality, and overall laying performance.

## Introduction

Conventional feeding practices for laying hens typically involve the provision of a complete diet with unrestricted access to food (*ad libitum*) (Hwang et al., 2025). The diet is commonly presented in the form of pellets or crushed feed, thereby leading to the regulation of the hens' food intake primarily by their energy requirements and the presentation form of the feed.

Within advanced poultry farming methodologies, the hens cannot adjust their consumption in alignment with their physiological necessities and production requisites. This often results in an overconsumption of nutrients, such as calcium, which holds a pivotal role in the formation of eggshells (Molnár et al., 2018a). The ultimate goal of egg production in hens is to attain specific productivity targets, including prolonging the production cycle to achieve an average of 500 eggs in 100 weeks. To achieve this objective, it is imperative to conduct studies and research endeavors focused on enhancing the quality of eggshells (Galea et al., 2015).

The eggshell serves a critical function by offering mechanical protection against harm and creating an appropriate environment for gas exchange during embryonic development. Furthermore, the shell serves as a distinctive "wrapping" for eggs destined for human consumption. Comprised predominantly of calcium carbonate, approximately 97 %, calcium stands as a vital mineral in the shell formation process, being included in the dietary regimen of laying hens. As delineated by Hunton (2005), in the digestive system of the hen, ionized calcium gets assimilated and subsequently incorporated in the shell gland. This process culminates in a notable turnover of calcium in the bloodstream, which can occur up to 100 times within a 24 h timeframe.

Research findings suggest that birds, in a general context, possess the ability to opt for their nourishment to meet their growth and maintenance requirements (Barkley et al., 2004; Cruz et al., 2005; Henuk and Dingle, 2002; Keshavarz, 1998a; Leeson, Summers, 1978; Syafwan et al., 2012). Free-choice feeding systems enable birds to choose their sustenance based on their maintenance or production needs, thereby offering advantages over traditional feeding methods (Oliveira, 1999).

The endeavor to formulate diets aligning with the optimal requisites for hens has been an ongoing pursuit aimed at augmenting productivity and product excellence, alongside exploring the feeding conduct of these creatures (Clark et al., 2019). The concept of split feeding revolves around the provision of diverse feed types throughout the day, tailoring the diet to the hen's metabolic needs at different times of the day, primarily to enhance the quality of eggshells. By administering specific quantities of calcium at varied intervals during the day, the diet aligns more closely with the hen's requirements throughout the diurnal cycle, accounting for metabolic fluctuations. This approach aids in curbing bone utilization and consistently enhancing the quality of eggshells during the productive phase (Dacke et al., 1993), especially for hens approaching the culmination of their laying period (Galea, 2015). This review examines the physiological rationale and practical outcomes of split feeding in laying hens, with a specific focus on dietary calcium intake and its impact on eggshell quality.

## Egg development and feeding behavior of laying hens

Hens can lay an egg every 24-26 hours due to the presence of various follicles in their ovaries. These follicles, during the reproductive phase, exhibit a range of sizes that are organized based on their quality, starting from small and underdeveloped to large and capable of ovulation (Rutz et al., 2005). The specific follicle undergoing ovulation is denoted as the yolk. After the follicular membrane ruptures, the yolk is expelled from the ovary and delicately positioned in the infundibulum, which serves as the starting point of the oviduct.

Yolk development is a continuous process that takes place throughout the day, typically commencing around six days before ovulation. The progression of yolk formation advances through different stages, which are constrained by the duration the ovum remains within the uterus. A notable constraint in hens is their inability to significantly enhance shell thickness concerning the size of the egg, a factor that can be linked to calcium metabolism (Chah and Moran, 1985).

Studies have shown that hens provided with supplementary calcium tend to selectively opt for these components during the eggshell formation stage, thereby enhancing the quality of the shell. The assessment of calcium levels for laying hens is commonly carried out by analyzing the calcium content present in the shell and subsequently computing the daily intake based on a retention rate, typically ranging between 60 % to 70 %. Waldroup and Hellwin (2000) discovered that hens offered calcium separately from other ingredients displayed fluctuations in intake levels throughout the day, a pattern that is regulated by the process of shell formation.

The absorption of calcium in hens takes place in the duodenum and jejunum segments of the gastrointestinal tract, following which it is transported to the vascular system, acting as both the carrier and reservoir for calcium (Etches, 1987). Notably, not all calcium can be absorbed, with some being excreted. In the context of eggshell formation, calcium is sourced from dietary inputs consumed during the day, primarily ingested towards the conclusion of the photo phase (light period) (Etches, 1987).

The magnum region of the oviduct is responsible for the production of albumen and operates continuously throughout the day. Proteins that are present are generated by tubular glands, subsequently discharged, and then attached to the ovum as it traverses through the magnum. Following this stage, the process of protein storage is initiated until the subsequent ovulation cycle (Edwards et al., 1976).

Researchers such as Hiramoto et al. (1990) have underscored that both the oviduct and the liver in laying hens play pivotal roles in synthesizing proteins in larger quantities compared to any other analyzed tissue. Owing to the swift process of albumen formation, the synthesis of proteins in tissues like the oviduct may exhibit variations throughout the day. In contrast, the synthesis of liver proteins remains consistent irrespective of the phase of egg formation.

**Fig. 1:**
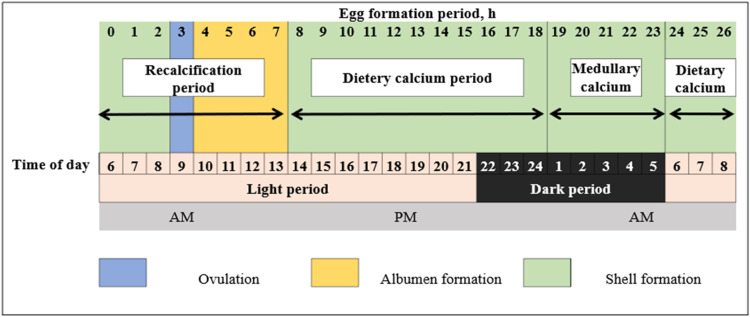
Stages of egg formation.

In Fig. 1, the various stages of egg formation are depicted, with a particular emphasis on feeding behavior associated with calcium intake. An evident disparity in consumption levels is noticeable during different times of the day, with the lowest calcium requirements observed in the morning and a notable surge in demand commencing at 4 p.m. This consumption pattern was evidenced by Mongin and Sauver (1974), who observed a considerable rise in intake between 4 p.m. and 8 p.m. when hens were supplemented with additional calcium from oyster shells in their diet. Chah and Moran (1985) further corroborated this behavior, indicating that when given a choice of calcium sources, laying hens exhibit specific consumption patterns, generally in smaller quantities. The graph vividly demonstrates the surplus of calcium available in the morning with a single feed, falling short of meeting the elevated demands of the hens later in the day.

It is widely accepted within the scientific community that the feeding behaviors of laying hens are substantially affected by the process of egg formation (Choi et al., 2004). Observations reveal heightened feed intake on days when egg formation is underway, as opposed to days when there is no egg formation occurring (Morris and Taylor, 1967). Taylor (1970) posits that the intake of feed during egg formation is primarily driven by calcium requirements rather than energy needs. Additionally, Hughes (1972) has pointed out that the intake of calcium is regulated on an hourly basis by the demands of egg formation.

Laying hens tend to consume approximately 2 % more feed on days when they are laying eggs compared to days when they are not laying eggs, as noted by Roland et al. (1972). According to the findings of Duncan and Hughes (1975), hens exhibit the capacity to self-regulate their feed intake, displaying decreased consumption levels during the period of luteinizing hormone release and an increase in feed intake during ovulation.

The requirements for amino acids in laying hens remain stable throughout the day, yet these demands can be impacted by the process of oviposition. Sqoibb (1966) emphasized that the amino acids necessary for the synthesis of proteins in the egg and shell membranes result in a significant release of free amino acids into the bloodstream, occurring within the initial five hours following oviposition. Furthermore, Grigoriev and Shevenko (1978) propose that there are fluctuations in the rates of amino acid absorption by the magnum region of the oviduct at different times of the day, coupled with varying rates of protein synthesis in this particular segment.

## Supply of feed to laying hens, main effects on production and nutrition

It is widely acknowledged that chickens possess a remarkable ability to selectively consume food particles, such as grains of corn, protein concentrates, and even limestone particles, to meet their daily nutritional requirements. Consequently, Robinson (1985) proposed a feeding system for chickens known as "bulk feeding," wherein a single diet is supplied throughout the day. This diet mainly comprises starch-rich grains, protein concentrates, and granulated limestone, thus encompassing a range of particle sizes.

By implementing this strategy, it becomes possible to simultaneously distribute essential nutrients within a single diet, thereby facilitating the separation of grains and enabling precise regulation of the intake of each component of the diet. In addition to this approach, sequential feeding is another method in which two distinct diets are provided throughout the day. These diets differ in terms of energy content, protein composition, and calcium levels. The objective of this technique is to ensure that specific nutrients are available at strategic moments during the process of eggshell formation (Umar Faruk et al., 2010).

Laying hens can be raised using various feeding systems, which can differ based on the breeder's expertise, the rearing system employed, and the types of food sources and distribution methods utilized. These systems may include providing complete and dry diets to hens ad libitum, offering pellets or crumbled feed to hens ad libitum, incorporating whole grains into complete diets, supplying restricted amounts of complete diets, or allowing hens to engage in grazing activities (Henuk and Dingle, 2002).

In laying hen rearing, complete diets remain the predominant choice due to the ease of management within the selected rearing system. Although other strategies such as crumbled feed offer benefits, particularly in terms of diet uniformity, they can be costlier due to the grinding, mixing, and labor involved in their preparation (Henuk and Dingle, 2002).

Significant genetic advancements in laying hens have led to alterations in various crucial physiological aspects, particularly those related to uniformity. Consequently, ensuring appropriate nutrition, alongside suitable health, welfare, and management practices, becomes vital to enable the hens to fully express their genetic productivity potential.

A significant proportion of the nutritional requirements is associated with the productive responses of laying hens, which are influenced by factors such as immunity, health, age, and nutrient interactions. These factors can impact digestibility and compromise hen performance (Araujo et al., 2014; Baião and Lucio, 2005).

To determine the daily metabolizable energy requirements of laying hens, various variables were taken into account, including body weight, weight gain, egg mass production, and the ambient temperature of the production environment, with temperature being a key factor in the hens' feeding process (Rostagno et al., 2017).

During peak production, hens generally consume more feed in terms of energy; however, they possess the ability to precisely adjust their energy needs by modifying their consumption (Leeson and Summers, 2005). In general, nutrient levels in the diet tend to decrease over time, except for calcium, as its availability does not diminish to meet the requirements for eggshell quality.

Therefore, there is an increasing focus on identifying management and nutritional strategies that can extend the productive lifespan of laying hens. Some researchers suggest forced molting as a method to rejuvenate the reproductive system and increase productivity in subsequent laying cycles (Park et al., 2004). However, Para-Silva-Mendonça et al. (2015) argue that fasting-based forced molting is a stressful practice that raises serious ethical concerns regarding animal welfare. In line with these concerns, current international welfare guidelines—such as the European Union Directive 98/58/EC—implicitly prohibit forced molting practices involving severe feed restriction and prolonged periods of darkness, as these methods are considered detrimental to animal welfare (Eurogroup for Animals, 2022). As an alternative, non-fasting molting protocols have been proposed to improve production efficiency while ensuring compliance with animal welfare standards (Lei et al., 2023).

High levels of calcium in the diet of laying hens can inhibit their feed intake (Hurwitz et al., 1969). On the other hand, calcium deficiency can lead to a reduction in their feed intake (Rolans, 1973; Bar, 2009). Hughes (1972) demonstrates that laying hens consume calcium in response to their physiologically stored reserves and possess the ability to anticipate a future deficiency of this mineral by detecting hormonal changes in their blood.

**Fig. 2:**
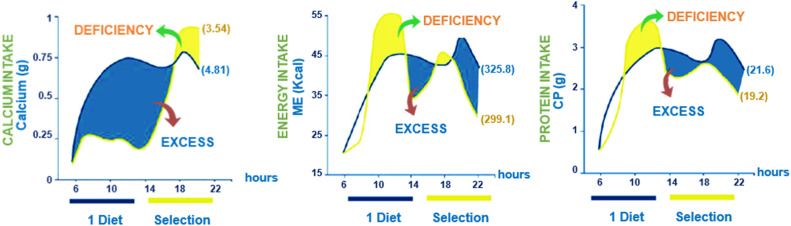
Consumption of calcium, energy, and protein by laying hens at various times throughout the day (Rech and Lora, 2022).

In an investigation conducted by Chah (1972), hens that were similar in age and subjected to uniform conditions were segregated into two distinct categories. On one hand, a collection of hens were allowed access to a solo feeder, whereas on the other hand, another collection had the advantage of using three distinct feeders. These feeders contained varying types of feed; one was abundant in energy, another in protein, and the third in calcium. The outcomes of the study exhibited variances in the behavioral patterns of the birds, as well as disparities in their consumption of nutrients and energy (Refer to Fig. 2).

Analyzing Fig. 2, we observe that hens exhibit varied feeding behaviors throughout the hours, with peaks in energy consumption during the early morning. This morning energy requirement is not met by a single diet, which forces the birds to consume more feed in the afternoon, primarily to meet their calcium needs. Consequently, protein intake is also elevated in the afternoon since the morning supply is insufficient. The comparison between the single diet and free-choice feeding reveals a marked difference in calcium intake, with a peak in intake in the late afternoon when a single diet is provided. By allowing choice, the hens demonstrated an ability to self-regulate, consuming fewer nutrients overall and optimizing egg production. 

**Fig. 3:**
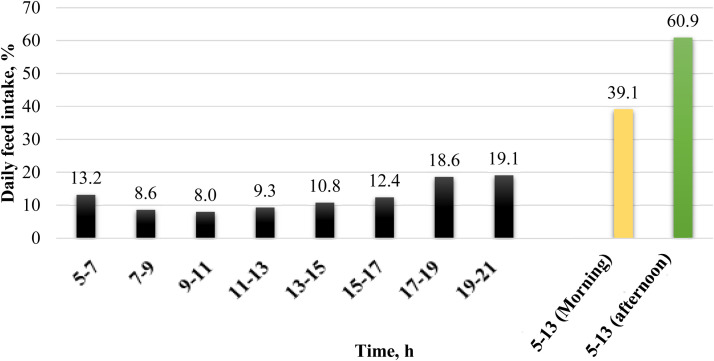
Feed intake of laying hens during the feeding cycle (Keshavarz, 1998a).

Understanding the foraging behavior of avian species transcends mere physiological scrutiny. It has been noted, as illustrated by Keshawarz (1998a) in his controlled trial, a predilection for feed intake in the later part of the day, substantiated by the increased proportion of intake during this period (Fig. 3). The variability in food consumption by egg-laying poultry throughout the diurnal cycle underscores the necessity for a more accurate and flexible dietary regime. A singular feeding schedule is inadequate in addressing fluctuations in nutritional demands, thereby compromising the productivity and well-being of the poultry.

## Self-selection of feed

Henuk and Dingle (2002) evaluated the free-choice technique for laying hens, which allows for the selection of three primary types of food. The findings revealed improved performance levels and reduced feeding costs. It can be concluded that when allowed to choose, birds can consume the most appropriate food for their physiological state and production level. However, it is important to consider that the nature and method of offering food can significantly impact the consumption patterns of laying hens.

In the wild, birds generally have a range of ingredients to compose their diet. In this context, domestic birds can choose ingredients that favor their nutrition. Modern domestic birds demonstrate this ability to adjust their feeding behavior in a natural environment to meet the basic requirements of growth, production, and reproduction (Cruz et al., 2005; Henuk and Dingle, 2002).

In situations where choice is an opportunity, birds can regulate the intake of a wide variety of foods. Visual stimulation is considered crucial in this process, with food preferences easily identified (Fraser and Broom, 1997).

Studies have shown that laying hens have superior visual capabilities compared to pigs, thus heavily relying on vision in food search, emphasizing again that particle size is fundamental in the feeding process of birds (Henuk and Dingle, 2002).

After investigating possible practical and economic advantages of self-selection for chickens, Henuk and Dingle (2002) affirmed that birds can choose their diet from a variety of foods, thus ensuring the satisfaction of their nutritional needs. Additionally, the same authors emphasized that self-selection eliminates the need to mix ingredients, resulting in energy savings. Furthermore, they considered that complete mixed diets are not essential, which also saves on the purchase of more expensive foods, allowing for the use of regional ingredients.

## Split feeding

Commonly adopted in various laying hen production systems, the practice of supplying a single diet may not be an optimal nutritional strategy for egg production (Keshavarz, 1998b). An alternative approach, known as Split Feeding, involves dividing the feeding regimen into two distinct diets for morning and afternoon. The composition of the morning diet is tailored to meet the specific nutritional requirements during the early stages of egg formation, with a particular focus on energy and protein (Jahan et al., 2024). In contrast, the afternoon diet is designed to support eggshell formation, particularly regarding calcium.

According to Molnár et al. (2018b), the primary aim of split feeding is to enhance egg quality by strategically adjusting nutrient levels—particularly calcium and phosphorus—between the morning and afternoon diets. This feeding strategy allows for a more efficient use of nutrients by supplying them at the time they are most required for egg formation, thereby potentially reducing the overall dietary requirements for amino acids, calcium, and phosphorus.

In conventional rearing practices, hens are provided with the same diet throughout the day, resulting in a consistent calcium intake. However, this may not be the most efficient approach, as the demand for calcium varies at different times of the day (Hwang et al., 2025). Supplying calcium outside of the appropriate time can lead to nutrient wastage in laying hen feed (Molnár et al., 2018b).

During the initial stages of egg formation, which include ovulation and albumen formation that occur within the first 5-6 hours, the demand for calcium is approximately 40 % of the available calcium. As the shell formation process takes place, this demand increases to 70-80 % (Hurwitz and Bar, 1965).

Molnár et al. (2018b) underscore the necessity of implementing a novel feeding system for production and laying hens throughout their productive lifespan to optimize nutritional efficiency. The split feeding method involves the provision of different nutrients at specific times of the day, in the morning and afternoon, with the main goal of preserving the quality of eggshells. Calcium supply, mostly from limestone sources, is of primary objective of preserving eggshell quality.

Although limestone, in the form of calcium carbonate, is widely used in layer hen diets, alternative sources such as oyster shell meal, and mollusk shells have also been investigated (Faria et al., 2000; McLaughlan et al., 2014; Mako et al., 2017). However, limestone stands out as the preferred source in commercial systems due to its low cost, high availability, and practical effectiveness.

The supply of calcium, predominantly sourced from limestone, is of utmost importance in the nutritional process, and particle quality must be taken into account in both conventional diets and split feeding strategies. Fine and coarse limestone are the primary calcium sources employed in avian nutrition, differing not only in particle size but also in solubility.

Coarser limestone particles (> 0.8 mm) take longer to dissolve in the gizzard, resulting in a slower release of calcium. Conversely, fine particles, in powder form, provide readily available calcium for absorption (Zhang et al., 1997).

Molnár et al. (2018b) propose that the use of fine limestone may be a favorable strategy for morning feeding of hens, while the use of coarse limestone is recommended for the afternoon. The authors highlight the significance of further research in this area, considering that the morning diet promotes calcium absorption and its storage in the bones, while the afternoon diet ensures a sustained release of calcium to support eggshell quality overnight.

This feeding strategy aligns with the physiological mechanisms involved in calcium metabolism in laying hens. Efficient calcium absorption primarily occurs in the duodenum and jejunum and is mediated by transporters such as TRPV6 and Calbindin-D28K, whose expression is regulated by vitamin D₃ in its metabolically active form, 1,25-dihydroxycholecalciferol (1,25(OH)₂D₃) (Ribeiro et al., 2025). Once absorbed, calcium can be temporarily stored in bones and later mobilized—under hormonal regulation, especially by parathyroid hormone (PTH)—to meet the high demands of eggshell formation during the night. Calcitonin also contributes to this regulatory process by modulating serum calcium levels and preventing excessive bone resorption (Ribeiro et al., 2024a; Ribeiro et al., 2024b).

The morning diet should contain higher levels of proteins and energy compared to the afternoon diet, owing to the increased protein requirement for albumen formation. Additionally, the authors note that the adoption of this strategy leads to an improvement in eggshell quality.

## Impacts on activity and yields

Under optimal environmental conditions at around 22°C, the feed intake of laying hens is regulated based on their requirements for production and maintenance processes. According to NRC (1994), the intake of nutrients such as amino acids, minerals, and vitamins is controlled by the energy content of the diet.

Laying hens primarily adjust their intake based on the energy content of their feed. However, this system may lead to excessive energy intake, causing the animals to consume more than their daily needs for maintenance and egg production. This can result in metabolic disorders such as excessive body weight, which in turn increases the demand for energy for maintenance (Snetsinger and Zimmerman, 1974).

In laying hen production systems, feed supply costs can account for approximately 70 % of all operational costs. Minimizing waste caused by excessive nutrient intake is crucial for maintaining the production chain. Enhancing the birds' digestive capacity can enable the use of lower-quality feed, thereby further reducing operational costs (Adeyemo et al., 2017).

Research that encourages a reduction in feed intake without compromising the quality of eggs, particularly egg mass, contributes to increased profitability in this activity (Akter et al., 2018; Fairfull et al., 1984).

## Effects of split feeding on performance and eggshell quality

Holcombe et al. (1976) conducted two experiments intending to determine the ability of laying hens to regulate their phosphorus intake when offered diets containing varying levels of phosphorus. In the first experiment, 72-week-old Babcock B-300 hens were divided into four groups. The control group received a diet with 0.75 % phosphorus in both cups. The second group was given a diet with 0.19 % phosphorus in one cup and 0.46 % phosphorus in the other. The third group had the option to choose between a diet with 1.00 % phosphorus in one cup and a diet with 2.43 % phosphorus in the other. The fourth group could choose between a diet with 0.19 % phosphorus in one cup and a diet with 2.43 % phosphorus in the other. The second experiment involved the same food choices and 48-week-old birds. In both experiments, the hens regulated their phosphorus intake when presented with these options. The younger hens showed a significant increase in consumption of the diet with 0.46 % phosphorus compared to the diet with 0.19 % phosphorus. Both the older and younger hens in the 0.46 % phosphorus versus 0.19 % phosphorus group in both experiments maintained egg-specific gravity, egg weight, and egg production at levels comparable to the control group. The results of two-hour feed weighing indicated that laying hens in the 0.19 % phosphorus versus 2.43 % phosphorus range exhibited a peak in preferred phosphorus consumption at noon, followed by a sharp decline from afternoon to evening. Chah and Moran (1985) experimented with 67-week-old hens to determine if the hens would adjust their nutrient intake to accommodate egg formation and alter albumen. The birds were divided into two groups: one group received complete feed for 4 weeks, while the other group had free choice of a high-energy feed (8 % protein, 2,800 kcal ME/kg, 0.75 % Ca), a 50 % protein pellet (2,500 kcal ME/kg, 0.20 % Ca), and oyster shell flakes. The researchers did not observe any differences in production or body weight due to the feeding regimen. The resulting eggs had improved shell strength and increased thin internal albumen at the expense of thick albumen. The protein concentration of the outer thin, thick, and inner albumens remained constant, but the ovalbumin A1 constituent increased while conalbumin, ovalbumins A2, and A3 decreased. Therefore, the authors concluded that hens consume nutrients according to their needs for egg formation and, as a result, improve shell and albumen yield. However, these findings also highlight that nutritional adjustments may involve trade-offs in albumen composition, suggesting that optimizing one quality trait (e.g., shell strength) might come at the expense of another (e.g., albumen viscosity).

Lee and Ohh (2002) conducted two experiments intending to evaluate the effects of nutrient levels and feeding methods of fractionated diets on the performance of ISA Brown laying hens, feed cost, and eggshell quality. The experiments were carried out from 4:00 AM to 3:00 PM and from 3:00 PM to 9:00 PM. In Experiment I, the birds (30 to 38 weeks old) were divided into four treatment groups. The control treatment (C) involved feeding the birds a single conventional diet throughout the day. The split diet groups (T1, T2, and T3) were given diets with high energy/protein and low calcium content during the morning, and diets with low energy/protein and high calcium content during the afternoon. The daily consumption of metabolizable energy (ME) and crude protein (CP), as well as feed cost, were significantly reduced in the split diet groups compared to the control group. However, there were no significant differences in egg production per day, average egg weight, and daily feed intake among the different treatment groups. The split diet groups also showed significant improvements in feed conversion ratios for feed, ME, CP, and feed cost required per day and kilogram of egg mass. In Experiment II, the birds (50 to 58 weeks old) were divided into three treatment groups. The control group (C) was fed a single conventional diet throughout the day, while the split diet group (T1) received diets with high energy/protein and low calcium content during the morning and afternoon. The second treatment group (T2) was given a mixed diet in which the morning diets were in mash form and the afternoon diets were in pellet form. The daily feed intake and average egg weight were reduced in both T1 and T2 compared to the control group, while egg production per day was not affected by the feeding system. The daily consumption of ME and CP and the feed cost were also reduced in T1 and T2, and there were improvements in the conversion ratios of ME, CP, and feed cost required per egg. However, there were no significant differences in the feed conversion ratios for feed, ME, CP, and feed cost required per kilogram of egg mass compared to the control group. The eggshell quality in T1 and T2 was found to be improved compared to the other treatment groups. Therefore, the authors concluded that the introduction of fractionated diets for morning and afternoon feeding, as well as the feeding method of mixed diets from fractionated diets, could lead to reduced feed and nutrient consumption and feed cost per day or kilogram of egg mass. Additionally, the feeding method of mixed diets was found to be convenient and effective in terms of saving feed costs and improving eggshell quality.

To mitigate the impact of aging on eggshell quality in mature laying hens, Molnár et al. (2018b) conducted a study wherein six different dietary treatments were tested on hens aged between 75 and 92 weeks. The conventional treatment (T1) involved offering a diet consisting of a 50:50 ratio of fine limestone (FL) and coarse limestone (CL) during both the morning (M) and afternoon (A) feeding periods. In the fractionated treatments, the ratio of FL to CL varied, either 50:50 or 30:70, and the timing of administration (M/A) differed. The remaining treatments were as follows: T2 = 50FL-M:50CL-A; T3 = 50CL-M:50FL-A; T4 = 30FL-M:20FL-A+50CL-A; T5 = 30FL-M:70CL-A; and T6 = 0M:30FL-A+70CL-A. The results of the study led the authors to conclude that, within the split-feeding system, the optimal combination of morning and afternoon diets was one where the morning diet consisted solely of fine limestone and the evening diet solely of coarse limestone (T2). Both diets provided approximately 50 % of the total daily calcium intake. This particular diet was found to sustain eggshell-breaking strength and dynamic stiffness between 75 and 92 weeks. Decreasing the amount of calcium in the morning and increasing it in the afternoon did not result in any improvements in eggshell quality characteristics. Furthermore, the particle size or inclusion level of limestone did not have any notable impact on eggshell quality within the split-feeding system.

Molnár et al. (2018c) conducted a study involving brown laying hens to assess various split feeding systems to prolong the hens' productivity cycle and enhance shell quality. The researchers found that although the split feeding system did not effectively maintain shell quality, it did demonstrate some promise in increasing the relative weight of the shells. The authors also noted that implementing split feeding in the aviary system posed practical challenges, and the flock experienced health and welfare concerns both before and during the experiment, which had an impact on the overall performance and results of the study.

El-Razek et al. (2020) conducted a study to assess the quality and production parameters of Dandarawi laying hens during the late production period. The hens were divided into two groups: a control group that received a standard diet with optimal levels of energy, protein, and calcium throughout the day, and an experimental group that followed a split feeding system. The experimental group had access to two different diets throughout the day. From 6:00 AM to 6:00 PM, they were offered a diet high in energy and protein, but low in calcium. From 6:00 PM to 6:00 AM, they were given a diet low in energy and protein, but high in calcium. The daytime diet contained 10 % more energy, 23 % more protein, and 50 % less calcium than the nighttime diet. The researchers observed that this split feeding system led to a decrease in feed intake, an improvement in feed conversion ratio, a reduction in feeding costs, and an increase in egg mass.

**Fig. 4:**
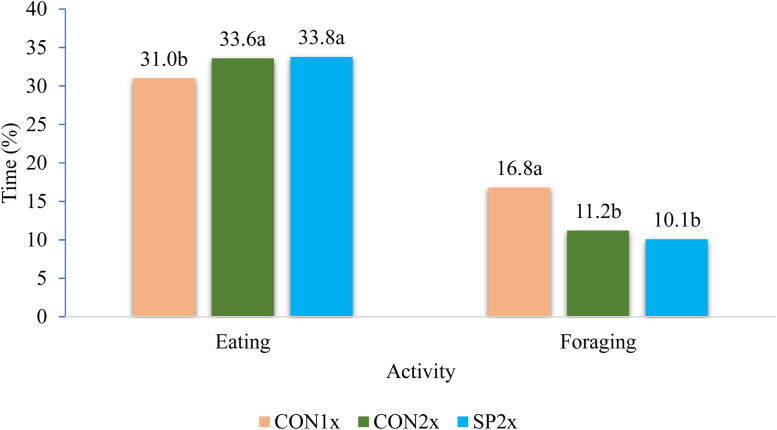
Effects of different feeding strategies and age on behavior (% of Time) (Van Emous and Mens, 2021).

Van Emous and Mens (2021) conducted an experiment employing three treatments to examine the effects of providing a standard diet twice a day or split feeding on broiler breeders aged 51 to 60 weeks. The study focused on productive performance, eggshell quality, incubation characteristics, and behavior. The three treatments were as follows: CON1x: a once-daily feeding of the standard breeder diet, with 100 % given at 07:30; CON2x: a twice-daily feeding of the standard breeder diet, with 50 % given at 07:30 and the remaining 50 % at 14:00; and SP2x: a twice-daily split feeding, with a specially formulated composition for the morning (07:30) and afternoon (14:00) diets. The morning diet was comparable in terms of energy to the control diet but had higher protein and phosphorus levels and lower calcium content. The afternoon diet, on the other hand, had lower energy, protein, and phosphorus levels, and higher calcium content compared to both the control and morning diets. No significant differences were observed in total egg production or other production parameters. Furthermore, eggshell quality and incubation characteristics were not affected by the different feeding strategies. However, the feeding strategies did have a significant impact on behavioral patterns. Feeding twice a day resulted in more time spent eating and sitting, and less time spent foraging and pecking objects, as compared to once-daily feeding (Fig. 4). Therefore, the authors concluded that feeding twice a day improves behavior and egg production while having no noticeable effects on eggshell quality and incubation characteristics.

**Fig. 5:**
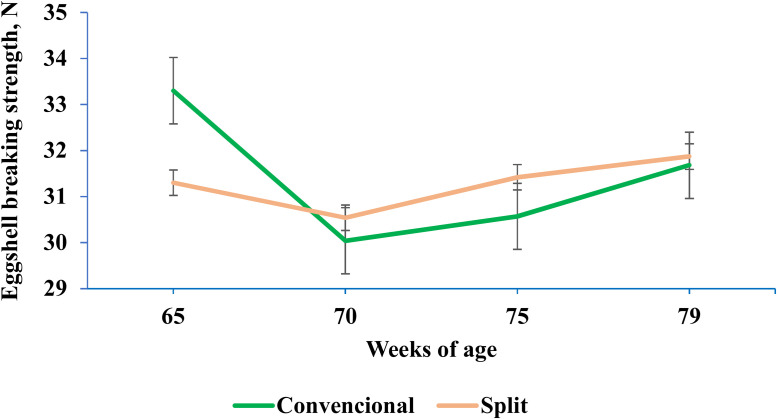
Interaction between feeding strategy and age on average eggshell breaking strength (N = Newtons). p-value = 0.04; SEM = 0.036. The p-value indicates the difference between feeding strategies at 65 weeks of age (Poudel et al., 2022).

Poudel et al. (2022) conducted a study involving broiler breeders aged between 60 and 79 weeks to assess the impact of different proportions of fine limestone (FL) and coarse limestone (CL) on egg production and quality. The experiment explored two methods of distributing the limestone feed: continuous (conventional) or only in the afternoon (split). A 2 × 4 factorial arrangement was employed, encompassing two feed distribution strategies (conventional and split) and four ratios of fine and coarse limestone (35:65, 25:75, 15:85, and 0:100 FL: CL). In total, eight feeding regimens were tested. Four regimens received the same diet in both the morning and afternoon, while the remaining four regimens received a diet lacking limestone in the morning, but with all the limestone incorporated in the afternoon. The authors observed that, during the later stages of egg laying, eggshell strength decreases, leading to an increase in the occurrence of soft-shelled and cracked eggs. However, the split feeding regimen effectively maintains eggshell strength in the later stages of laying (Fig. 5). Furthermore, the addition of coarse limestone exceeding 75 % negatively impacted tibia-breaking strength.

**Fig. 6:**
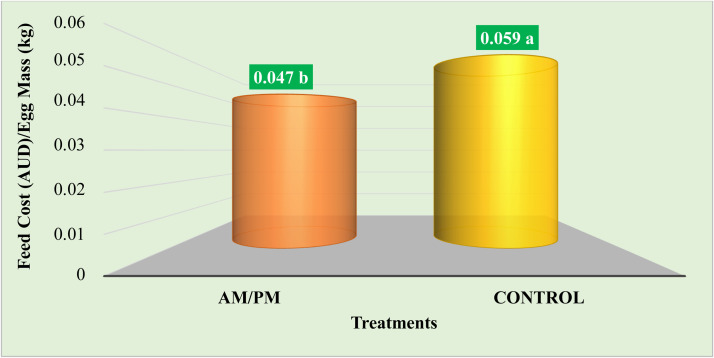
Cost–benefit analysis of the dietary treatments over 20 weeks of this study (Jahan et al., 2024).

To assess whether providing laying hens with diets tailored to their specific nutritional and physiological requirements at different times of the day, as opposed to a uniform diet throughout the day, is advantageous, Jahan et al. (2024) conducted a study involving Hy-Line Brown laying hens aged 34 to 53 weeks. The hens were divided into two groups: group 1 received a standard diet for laying hens continuously throughout the day (control), while group 2 received a morning (AM) diet from 8:00 AM to 4:00 PM and an evening (PM) diet from 4:00 PM to 8:00 AM (AM/PM). Overall, the findings indicated that the AM/PM treatment led to a 2.15 % increase in egg mass (60.4 vs. 59.1 g/bird/day) and an 8.34 % improvement in feed efficiency (2.231 vs. 2.436 kg of feed/kg of egg) compared to the control. Eggs from the AM/PM treatment exhibited a higher yolk color score, but no significant effects on egg quality were observed. Ileal digestible energy and digestible nitrogen coefficient were lower in hens on the AM/PM treatment compared to the control treatment (Table 1). However, the AM/PM treatment resulted in lower feeding costs per unit of egg mass compared to the control treatment (Fig. 6). Consequently, the authors concluded that the AM/PM feeding strategy demonstrated economic benefits.

**Table 1:** Effect of AM/PM feeding on nutrient digestibility at 53 weeks of age.

Treatments	Variables		
	IDE	IDEC	IDNC
AM/PM	2305 b	0.61 b	0.70 b
Control	2808 a	0.7 a	0.80 a
SEM	81.2	0.02	0.01
p-value	<0.001	0.008	<0.001

IDE: ileal digestible energy, IDEC: ileal digestible energy coefficient, IDNC: ileal digestible nitrogen coefficient. a,b Means within the rows with different suffixes are statistically different at the 5 % level of significance. Source: Adapted from Jahan et al. (2024).

In the study conducted by Hwang et al. (2025), the authors evaluated the impact of split feeding on laying hens by comparing a conventional single-diet approach with two split-feeding strategies (TRT1 and TRT2) that varied in nutrient composition between the morning and afternoon. The findings demonstrated that hens in the TRT1 group exhibited a significant reduction in the incidence of downgraded eggs, improved calcium and phosphorus digestibility, and lower ammonia (NH₃) emissions. Additionally, TRT1 achieved a 6 % reduction in diet costs without compromising performance. Based on these results, the authors concluded that aligning nutrient delivery with the physiological requirements of hens through split feeding may enhance production efficiency while simultaneously reducing the environmental footprint of egg production.

## Conclusions

By implementing split feeding, it becomes feasible to decrease feeding expenses via precision nutrition, while simultaneously enhancing egg quality. Nevertheless, the absence of studies about quail production utilizing this method necessitates further research to evaluate its suitability within this industry. Furthermore, the organization of feed factory logistics is crucial to ascertain the successful implementation of this approach on the farm.

## Declaration of competing interest

The authors declare no conflicts of interest.
